# OLDER-ADULT PERSONAL SUPPORT WORKERS’ CONTRIBUTIONS DURING THE COVID-19 EMERGENCY RESPONSE

**DOI:** 10.1093/geroni/igad104.1867

**Published:** 2023-12-21

**Authors:** Haorui Wu

**Affiliations:** Dalhousie University, Halifax, Nova Scotia, Canada

## Abstract

When COVID-19 devastated long-term care facilities across Canada, most public attention was turned to older adult residents/clients, rather than their service providers, especially the personal support workers (PSWs). Some PSWs are over 55 age and above; they featured dual roles in the COVID-19 settings: (1) they were a vulnerable group disproportionately impacted by COVID-19 and (2) they were essential workers providing support in the older-adult care facilities. Since current older-adult-driven research, practice, and policy have primarily focused on generalized assumptions of older adults as a vulnerable, passive, and dependent group rather than recognizing their diversity, expertise, assets, and experiences, this project aims to identify these older-adult PSWs’ contributions. This qualitative study invited 13 PSWs from older-adult care organizations in the Greater Toronto area of Canada to participate in in-depth interviews. This study uncovered three major contributions of older-adult PSWs within the individual-work-family triangulation. At the individual level, they bear the potentially high health risks of COVID-19, while supporting people around them. At the workplace level, they took on extra responsibilities to reduce the spread of the virus, dealing with the client’s/residents’ unfavourable behaviours and stress, and supporting their co-workers to maintain their organizations’ operations. At the family level, they worked overtime to compensate for their family members’ income loss and kept their family members at home to avoid infections. These outcomes inform the older adult research, practice, and policy by enhancing the appreciation of older adults’ diverse strengths and promoting older adults’ contributions in disaster settings.

